# Nutritionists’ Practices and Knowledge about the Risks of Alcohol Consumption during Pregnancy: An Israeli Survey

**DOI:** 10.3390/nu14091885

**Published:** 2022-04-29

**Authors:** Liat Hen-Herbst, Meital Ron El Levin, Yehuda Senecky, Sigal Frishman, Andrea Berger

**Affiliations:** 1 Department of Occupational Therapy, Ariel University, Ariel 4077000, Israel; 2 Women’s Hospital, Rabin Medical Center, Petach Tikva 4941492, Israel; meitalron@gmail.com; 3 Department of Pediatric Neurology and Child Development, Schneider Children’s Medical Center of Israel, Petach Tikva 4920235, Israel; senekimi@zahav.net.il; 4 Nutrition Department, Hospital Division, Clalit HMO, Petach Tikva 4941492, Israel; fsigal@clalit.org.il; 5 Department of Psychology and Zlotowski Center for Neuroscience, Ben-Gurion University of the Negev, Beer Sheva 8410501, Israel; andrea@bgu.ac.il

**Keywords:** alcohol consumption, (FASD) fetal alcohol spectrum disorder, pregnancy, prenatal alcohol exposure, preventive medicine

## Abstract

Fetal alcohol spectrum disorders (FASDs) are lifelong disabilities and the leading preventable cause of developmental disabilities. Antenatal care providers may influence pregnant women’s dietary practices and their awareness of the risks of alcohol consumption during pregnancy. This study aimed to assess nutritionists’ self-reported knowledge about the risks of drinking alcohol during pregnancy, professional practices in this respect, and self-perceived competence to assess and guide women about alcohol consumption during pregnancy in Israel. A sample of 526 professional nutritionists completed an anonymous online questionnaire. Results showed significant differences between the nutritionists’ knowledge and professional practices scores. About 349 (66.3%) of the sample agreed (to any degree) that they did not have enough knowledge to guide pregnant women regarding drinking alcohol. The number of years of experience, combined with self-perceived competence and the mean knowledge score, explained 18% of the variance in professional practices. Nutritionists and other health professionals may have a crucial role in preventing FASD and should prioritize appropriate screening for prenatal alcohol use. Eliminating alcohol consumption at any point in pregnancy would reduce the risk for FASDs.

## 1. Introduction

All gestational periods require good balance in order to interchange nutrients across the placenta, and thus are critical for correct fetal growth [1]. Optimal nutrition is essential for a fetus’s growth and development and can have long-term effects on the health of the mother and her infant [2]. Increasing evidence has shown that poor maternal nutrition can compromise the healthy development of a fetus. Women who drink alcohol before or during pregnancy, especially heavy drinkers, often have poor nutritional status [1]. Moreover, the toxic effects of alcohol or other drugs can damage maternal nutritional status, resulting in a lack of the essential nutrients required for the proper growth of the developing fetus and suboptimal health outcomes [3].

The critical influence of the maternal uterine environment on the offspring’s future health is called the developmental origins of health and disease hypothesis [4]. This hypothesis recognizes that exposure to certain environmental influences during critical developmental periods may have short- and long-term consequences for an individual’s health [5]. Combining this understanding with the evidence that alcohol is the most common drug for women of reproductive age [6], alcohol consumption among pregnant women is a major and serious public health concern [7]. Popova et al.’s [8] comprehensive systematic literature search and meta-analysis revealed that 10% to 15% of pregnant women in Canada and the United States consume alcohol. About 3% in both countries engage in binge drinking (four or more drinks in 2 h). In Israel, 67% of a sample of 802 pregnant women reported drinking any amount of alcohol in the 2 months prior to learning they were pregnant, and 12% reported drinking any amount of alcohol during their current pregnancy [9]. Furthermore, according to Senecky et al. [10], only a minority of women received guidance from a qualified professional regarding the risks of alcohol consumption during pregnancy. These high percentages demonstrate a need for studies examining knowledge and awareness of the risks of drinking alcohol close to recognition of, and during, pregnancy and whether professionals in public health have the knowledge, awareness, and practices to promote related education efforts.

Based on these indications, it is expected that prenatal alcohol exposure (PAE) will be considered a global health problem. However, its estimated prevalence varies worldwide—from 4.1% in Norway [11] and 7.3% in the United States [12] to 60.4% in Ireland [8]. Alcohol can negatively affect fetal development and cause a range of mental and physical disabilities, clinically termed fetal alcohol spectrum disorders (FASDs) [13], which create lifelong disabilities. Affected individuals can experience challenges in multiple areas, including executive functioning, learning, memory, attention, communication, and motor and social skills [14]. Recently published research from Canada showed that children with FASD were more likely to be diagnosed with health conditions (e.g., eye-related and skin conditions, chronic sinusitis, and ear infections) than the general population [15]. Epidemiological research has implied that FASD is a global problem [16] and the leading preventable cause of birth defects and developmental disabilities [17]. However, not every woman who drinks during pregnancy will deliver a child with FASD. Research has not yet delineated the pattern, amount, or critical period of PAE necessary to cause FASD [8]. Given these unknowns regarding structural or functional teratogenesis, many countries have modified clinical practice guidelines, advising health professionals to promote total alcohol avoidance throughout pregnancy [18]. The Israeli Ministry of Health [19] guidelines advise women to completely abstain from alcohol when trying to conceive and throughout their pregnancies. However, to date, the Ministry has no standard process to inform women of the risks.

Women who are not well-informed about the hazards of alcohol consumption during pregnancy may not comply with national recommendations or change their behaviors, attitudes, or levels of knowledge [20]. As the first points of contact, health care professionals, specifically nutritionists guiding pregnant women, can play a crucial role in preventing and identifying the risks of FASD [21]. Because decreasing alcohol consumption at any point in pregnancy reduces the risk of harm to the fetus, health professionals should prioritize appropriate screening for prenatal alcohol exposure [22]. In Israel, to date, there is no routine nutritional guidance for before pregnancy. However, the Ministry of Health guidelines for pregnancy suggest nutritional improvement 3 months before pregnancy as a part its “1000 Days Nutrition” program for better health outcomes for mothers and offspring until adulthood [23]. Currently, pregnant women, including those who are not at risk of developing special health conditions, have the rights and opportunity to ask for comprehensive guidelines detailing women’s nutritional needs from preconception and throughout pregnancy, given by nutritionists working in Israel’s health maintenance organizations. In addition, women at risk of developing diabetes and/or other conditions during pregnancy receive monthly consultations with a nutritionist. Hence, the nutritional knowledge and practices of pregnant women and their antenatal care providers may influence the dietary intake of pregnant women. Primary prevention measures, such as improving women’s nutritional knowledge, could be a cost-effective strategy for lowering the risk of adverse health outcomes for mothers and their infants [24].

However, to date, no previous studies have investigated the practices and knowledge of the risks of alcohol consumption during pregnancy among nutritionists in Israel. Our purpose in this study was to assess: (1) knowledge about the risks of drinking alcohol during pregnancy; (2) professional practices—to what extent nutritionists ask and advise pregnant women specifically about alcohol consumption; and (3) self-perceived competence to assess and guide women about alcohol consumption during pregnancy, among a national sample of professional nutritionists. Furthermore, we aimed to assess the relations among and between these variables and the sociodemographic and professional characteristics. Finally, we aimed to predict clinical practices based on professional experience, knowledge, and self-perceived competence.

## 2. Material and Methods

### 2.1. Study Design

This study used a cross-sectional survey that was conducted from February to June 2021. A sample of nutritionists volunteered to participate in our anonymous online survey. The Ethics Committee of the Psychology Department at Ben-Gurion University of the Negev approved the study (date of approval: 21 February 2020).

### 2.2. Participants 

A sample of 526 nutritionists (97% female) aged 18 to 71 years (*M* = 38.5 years) with 1 to 48 years of professional experience (*M* = 11.37 years, *SD* = 9.6) participated in this study (see Table 1). Most (87%) were born in Israel, and most (97%) studied nutrition in Israel. Nutritionists were recruited via emails that were circulated among nutrition service managers in various health care institutions around the country, and online through social media platforms (e.g., professional groups on Facebook). Participation was voluntary.

### 2.3. Survey Questionnaire

As part of the “call to action” to promote the prevention of alcohol consumption during pregnancy in Israel, we are conducting a set of studies to investigate the practices and knowledge of the risks across various health and allied health care provider groups. The results will help to inform targeted education programs that are tailored to address the gaps in knowledge and behavior. For this purpose, we developed an online survey with items that were considered common for all groups but were adaptable in content and wording for each group. In this study, the questionnaire took fewer than 10 min to complete and contained two parts.

#### 2.3.1. Part A: Sociodemographic and Professional Characteristics

Part A contained items regarding sociodemographic characteristics: gender, age, and country of birth. It also included professional measures: country of nutrition studies, number of years of professional experience, place of work, and whether the responder had completed special training on nutrition for pregnant women.

#### 2.3.2. Part B: Knowledge, Professional Practices, Self-Perceived Competence, and Specific Knowledge Regarding Binge Drinking

Part B was based on three questionnaires previously used to explore FASD-related knowledge, attitudes, and behaviors across professional and health care provider groups [25,26,27], however it was adapted for nutritionists in content and wording. Nutritionist representatives at several central health institutions piloted the online survey. After feedback, we modified some questions to improve the clarity of the survey and the descriptions of appropriate professional practices. Participants rated how much they agreed with each statement on a Likert scale from 1 (absolutely agree) to 5 (absolutely disagree).

Eleven items assessed knowledge of the effects of alcohol consumption during pregnancy; their reliability in assessing knowledge was α = 0.697. Five items assessed nutritionists’ practices during their meetings with pregnant women; their reliability in assessing professional practices was α = 0.702. Additionally, one item assessed nutritionists’ self-perceptions of their competence to assess and guide women about alcohol consumption during pregnancy. This item asked participants to rate their agreement with the statement, “I feel that I do not have enough knowledge to guide pregnant women regarding alcohol consumption during pregnancy”, and was analyzed separately. Finally, Item 17 addressed the importance of knowledge regarding the risks of binge drinking. It stated “Binge drinking (four or more drinks in about 2 h) is more dangerous to the fetus than is daily consumption of one alcoholic beverage”. While reviewing the results of our previous research [9] that assessed knowledge of the possible risks of PAE among a sample of Israeli pregnant women, we noticed that women were unaware of the increased risks associated with binge drinking, which called for clearer public health education about the risks and consequences of binge drinking for pregnant women. Therefore, we decided to analyze this issue separately among the nutritionists; we investigated this specific knowledge among those who could have the opportunity and power to educate pregnant women.

Eight of the questions were purposely worded in the opposite direction, and were reversed before analysis. In the final scale, higher ratings indicated a greater level of knowledge and self-perceived competence, and the use of more professional practices (see Table A1).

### 2.4. Data Analysis

We analyzed the data using SPSS software version 26 (IBM Corp., Armonk, NY, 2019), using descriptive statistics for the demographic and professional characteristics. The means and standard deviations were presented for continuous data and frequencies, and percentages were presented for categorical data. Intragroup comparisons were conducted with paired *t* tests. Associations between the sociodemographic characteristics, knowledge about the risks of consuming alcohol during pregnancy, and professional practices were assessed using chi-square tests for discrete variables, independent sample *t* tests, one-way analysis of variances for continuous and discrete variables, and Spearman correlations for ordinal variables. Stepwise linear regression was used to identify possible predictors for the nutritionists’ reports about their professional practices from the following candidate variables: knowledge; self-perception of competence; age; and years of experience, with a cutoff of *p* < 0.05.

## 3. Results

### 3.1. Demographic and Professional Characteristics

Table 1 shows the participants’ demographic and professional characteristics.

### 3.2. Descriptive Results of Knowledge, Professional Practices, Self-Perceived Competence, and Knowledge Regarding Binge Drinking

Table 2 shows the descriptive statistics for the measures.

### 3.3. Associations between Knowledge about the Risks of Alcohol Consumption during Pregnancy, Professional Practices, Knowledge Regarding the Risks of Binge Drinking and Self-Perceived Competence, and between Demographic and Professional Characteristics

Table 3 shows the associations between the scale measures and the demographic and professional characteristics. No correlations were found between the mean score of general knowledge or the measure of specific knowledge regarding the risks of binge drinking during pregnancy and all demographic and professional characteristics. However, the mean score of professional practices was significantly associated with age and years of experience, meaning that older nutritionists with more years of professional experience raised the topic more often and asked pregnant women about alcohol consumption more often. Finally, the results showed relatively high agreement with the statement that they “do not have enough knowledge to guide pregnant women regarding alcohol consumption during pregnancy” among nutritionists who had not been specially trained to guide pregnant women (Table 3).

### 3.4. Associations between Scale Measures

Table 4 shows the associations between the four scale measures. We found a small positive correlation between the mean scores for knowledge and professional practices. Furthermore, we found significant correlations between self-perceived competence and the mean scores for knowledge and professional practices. Finally, we found an interesting positive correlation between the score of specific knowledge about the risks of binge drinking during pregnancy and the mean professional practices score, such that nutritionists with better specific knowledge about the risks of binge drinking during pregnancy were more inclined to ask and guide pregnant women regarding alcohol consumption (Table 4).

### 3.5. Differences between Knowledge and Professional Practices Mean Scores

The results of the paired *t* tests used to analyze gaps between the knowledge and professional practices mean scores showed a significant difference between knowledge and actual performance scores, *t* (525) = 12.86, *p* = 0.000, d = 0.73.

### 3.6. Knowledge Regarding the Risks of Binge Drinking

As mentioned in Section 2, we analyzed Item 17 separately. Of the 526 participants, 37 did not complete this item. Among those who answered, only 42 (8%) absolutely agreed, 68 (12.9%) very much agreed, 164 (31.1%) moderately agreed, 83 (15.7%) slightly agreed, and 134 (25.4%) absolutely did not agree with the statement—meaning that more than 40% of our sample did not know of the increased risks associated with binge drinking. The mean score for this item was significantly lower than the mean score for the assessed knowledge scale, *t* (490) = 10.81, *p* = 0.000, d = 0.69.

### 3.7. Self-Perceived Competence

Of the 526 respondents, 44 (8.3%) absolutely agreed, 67 (12.7%) very much agreed, 111 (21%) moderately agreed, 115 (21.8%) slightly agreed, and only 189 (35.8%) absolutely did not agree that they had insufficient knowledge to guide pregnant women regarding alcohol drinking.

**Figure 1:**
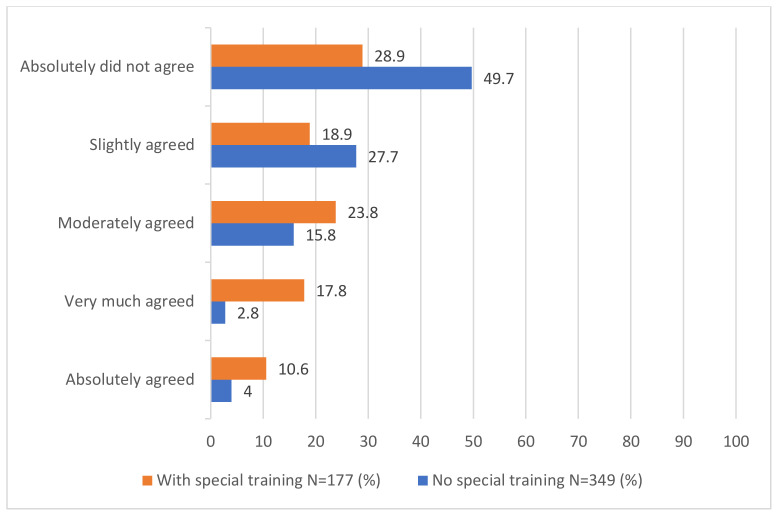
Frequencies of answers to Item 12 among participants with special training compared to participants without special training.

The results showed relatively high agreement with this statement among nutritionists who had not been specially trained to guide pregnant women (See Figure 1). The mean score for those who had special training was 4.16 (*SD* = 1.05), and the mean score for those who did not have special training was 3.38 (*SD* = 1.35), *t (525)* = 7.35, *p* = 0.000, d = 0.62.

### 3.8. Prediction of Professional Practices by Years of Experience, Self-Perceived Competence, and Knowledge Scores

The results suggested three models (Table 5). The first model indicated that years of experience explained 8% of the variance in professional practices (R^2^ = 0.08), *F*(1, 524) = 45.49, *p* = 0.000. The second indicated that the number of years of experience combined with self-perceived competence explained 15% of the variance in professional practices (*R*^2^ = 0.15), *F*(1, 523) = 43.83, *p* = 0.000. Finally, the third model indicated that the number of years of experience combined with self-perceived competence and the mean knowledge score explained 18% of variance in professional practices (R^2^ = 0.18), *F*(1, 522) = 37.42, *p* = 0.000.

## 4. Discussion

Although preventable, FASD burdens the affected patients, families, and society. Unfortunately, the literature suggests that many nutritionists and health care professionals do not perform screenings or interventions to reduce PAE as a part of their antenatal consultations [28,29]. This study evaluated the professional practices and knowledge of the risks of alcohol consumption during pregnancy among a sample of nutritionists in Israel. This research is one link in a “call to action” for every health care professional who has the position, education, and accountability to counsel women about leading healthy lifestyles during pregnancy and their relationships with short- and long-term health effects for the mothers and their infants. In general, the nutritionists in this study knew about the risks of alcohol consumption in pregnancy. We found no significant differences in the mean knowledge score by sociodemographic or professional characteristics. Unfortunately, the expectation that nutritionists with special training to guide pregnant women would score higher on knowledge did not materialize. Still, this result might be considered as a positive, promising finding suggesting that Israeli nutritionists know the potentially damaging effects of alcohol consumption during pregnancy and could educate and guide pregnant women.

Hence, a deeper look at our results—specifically those of the item explicitly asking about binge drinking—suggests that the nutritionists’ knowledge in this respect might not be deep enough. Considering that 7% of the participants did not answer this item and that, among those who did answer the item, the mean score was significantly lower than the mean score for the more general knowledge scale, it appears that knowledge regarding the risks of binge drinking is less common. These results raise questions about the depth of nutritionists’ knowledge and whether their knowledge allows them to professionally guide women’s alcohol drinking habits before and during pregnancy. Further studies incorporating broad subject knowledge must be conducted in order to answer these questions.

In our study, the mean knowledge score was significantly higher than the mean professional practices score. Moreover, our survey indicates that with their general knowledge about the risks of alcohol consumption during pregnancy, few nutritionists routinely ask pregnant women about their alcohol drinking habits before and during pregnancy as a part of their professional practices. This result is consistent with reports in other countries and professions (e.g., [30,31]).

According to Chiodo et al. [32], providers who did not fully understand the effects of alcohol consumption during pregnancy often did not feel prepared enough to educate their pregnant patients about the risks of prenatal alcohol consumption. They conducted their study among 581 nurses and midwives in the United States. They found that patients’ denial/resistance to treatment, time limitations, and patient sensitivity to screening were the most influential perceived barriers to screening pregnant women’s alcohol use. Other studies showed commonly reported barriers for primary care providers regarding screening for alcohol consumption during antenatal care, including a lack of understanding about the consequences of alcohol exposure on the fetus and insufficient knowledge of how to screen effectively (e.g., [28,30]). No previous studies have investigated the perceived barriers to antenatal alcohol screening among health professionals in Israel. Our findings point to a gap between knowledge and performance, and highlight the need for future studies to test the perceived barriers.

Our results emphasize the importance of self-perceived competence as a key factor associated with knowledge and professional practices. Together with professional experience and knowledge, self-perceived competence predicted professional practices. Previous research among other health providers also noted a perceived lack of knowledge and skill to screen and support pregnant women (e.g., [28,33]). For the first time (to the best of our knowledge), our results highlight that nutritionists perceive the same lack of competence. We found significant differences in self-perceived competence between nutritionists who had participated in special professional training for guiding pregnant women and those who had not. This result aligns with previous results [34] reporting community health and social care practitioners’ improved confidence, perceived competence, and intent to use new professional tools to help change health behaviors. These improvements occurred following involvement in a professional course that focused on developing practical skills and implementing behavioral science. These results demonstrate the importance of ongoing specific training in order to increase health professionals’ confidence in discussing alcohol consumption and their ability to sensitively pose appropriate questions. They emphasize that specific education and professional training are essential for promoting self-perceived competence among health professionals. Additionally, understanding the psychological determinants of change is necessary to ascertain and maximize the usefulness of such training in practice. Further work is required to generate feasible, acceptable ways of evaluating the impact of such training on actual practice behaviors.

Screening, or even consistently asking pregnant women about alcohol use, increases the awareness of, and alters behaviors towards, alcohol use in pregnancy [35]. Studies exploring the perspectives of pregnant women show that they find it acceptable to screen for alcohol use during pregnancy [36,37]. Our results emphasize the importance for every prenatal care provider—especially nutritionists—to maximize the trust relationship, ensure a judgment-free environment, and ask pregnant women about drinking alcohol in the 2 to 3 months before they recognize their pregnancy and during their pregnancy. Based on our results and shared evidence from previous studies, policymakers should be responsible for rechecking the content regarding pregnant women in nutritionists’ training programs. Moreover, they should develop new programs that aim to increase knowledge and provide tools to reduce the gap between knowledge and professional practices. To promote these aims, future efforts could include practice or willingness to provide a brief intervention or referral for women identified as having consumed alcohol during pregnancy andcomparisons to other topics that nutritionists routinely address with pregnant women (e.g., mercury or lead exposure and positive habits, such as eating green leafy vegetables or folic acid intake).

Several limitations should be acknowledged regarding this research. Firstly, because participation was voluntary and online, we were restricted in the number of items that we could include in the survey. This restriction prevented the possibility of including enough items to differentiate among knowledge domains. Moreover, only one item assessed self-perceived competence, and more items may be needed to assess this crucial measure in future research. Lastly, as with any survey, we relied on the participants’ sincerity and willingness to provide truthful responses in order to limit possible bias.

Despite these limitations, it is important to mention that this is the first Israeli study to examine nutritionists’ knowledge of the risks of drinking alcohol during pregnancy, professional practices in this respect, and self-perceived competence to assess and guide women about alcohol consumption during pregnancy.

## Figures and Tables

**Table 1:** Participants’ demographic and professional characteristics.

Characteristic	Mean (*SD*)	Range	Frequency (%)
**Age** (year)	38.5 (9.4)	18–71	
**Country of birth**			
Israel			458 (87)
Other			68 (13)
**Professional experience** (year)	11.37 (9.6)	1–48	
**Country of nutrition studies**			
Israel			510 (97)
Other			16 (3)
**Place of work**			
Health maintenance organization			217 (41)
Hospital/medical center			147 (28)
Freelancer/self-employed			162 (31)
**Special training on nutrition for pregnant women**			
Yes			176 (33.5)
No			350 (66.5)

Note: Sociodemographic and Professional Characteristics are in bold.

**Table 2:** Descriptive statistics for scale measures.

Measure	Mean (*SD*)	Range
General knowledge	4.07 (0.44)	2.50–5.00
Professional practices	3.55 (0.90)	1.00–5.00
Self-perceived competence	3.64 (1.30)	1.00–5.00
Specific knowledge regarding binge drinking	3.41 (1.26)	1.00–5.00

**Table 3:** Associations between scale measures and demographic and professional characteristics.

	Scale Measure	General Knowledge	Professional Practices	Self-Perceived Competence	Specific Knowledge Regarding Binge Drinking
Demographic and Professional Characteristic					
Ordinal variables (*r*)					
Age		−0.36	0.265 ***	−0.01	0.04
Years of experience		−0.27	0.283 ***	0.23	0.18
Discrete variables (χ^2^)					
Place of birth		31.78	31.77	4.66	2.34
Place of work		141.88	76.65	82.12	91.77
Place of nutrition studies		29.34	12.49	3.45	2.99
Special training		40.25	35.71	48.55 ***	13.76

*** *p* < 0.001.

**Table 4:** Association between scale measures.

	General Knowledge	Professional Practices	Self-Perceived Competence	Specific Knowledge Regarding Binge Drinking
General knowledge		0.23 ***	0.38 ***	−0.059
Professional practices	0.23 ***		0.27 ***	0.12 **
Self-perceived competence	0.38 ***	0.27 ***		−0.046
Specific knowledge regarding binge drinking	−0.059	0.12 **	−0.046	

** *p* < 0.01; *** *p* < 0.001.

**Table 5:** Prediction of professional practices.

Model	Variable	Unstandardized Coefficient		Unstandardized Coefficient	*t*	*p*
		*B*	*SE*	(β)		
1	Years of experience	0.027	0.004	0.283	6.74	0.000
2	Years of experience	0.026	0.004	0.277	6.86	0.000
	Self-perceived competence	0.187	0.028	0.267	6.62	0.000
3	Years of experience	0.027	0.004	0.288	7.23	0.000
	Self-perceived competence	0.140	0.030	0.200	4.66	0.000
	Knowledge	0.365	0.090	0.175	4.06	0.000

Note: The variable, “special training” was included in the multivariate analysis but was automatically dropped from the models.

## Data Availability

The data presented in this study are available on request from the corresponding author. The data are not publicly available due to ethical restrictions.

## Appendix A

Consequences of Alcohol Consumption During Pregnancy and Its Dangers (Translated from Hebrew to English).

Please read the statements below and choose the answer that best expresses your degree of agreement with each statement:

**Table A1:** The survey Questionnaire.

	Statement	Absolutely Agree	Very Much Agree	Moderately Agree	Slightly Agree	Absolutely Do Not Agree	Scale Measure
1	It is okay for a pregnant woman to drink an alcoholic beverage from time to time.						General knowledge
2	The issue of alcohol drinking habits will only be discussed if the pregnant woman raises it.						Professional practices
3	A person can recover from fetal alcohol syndrome.						General knowledge
4	Fetal alcohol syndrome is irrelevant to my professional job.						Professional practices
5	The Israeli Ministry of Health recommends completely avoiding drinking alcohol during pregnancy (including during Kiddush, a one-time social event, etc.)						General knowledge
6	People with fetal alcohol syndrome have brain damage.						General knowledge
7	Children with fetal alcohol syndrome may have growth retardation.						General knowledge
8	Fetal alcohol syndrome may impact the person for their entire life.						General knowledge
9	Women who are planning a pregnancy should absolutely avoid alcohol consumption.						General knowledge
10	I consistently ask pregnant women accurately and in detail about the amounts of alcohol they consume.						Professional practices
11	Drinking one alcoholic beverage during pregnancy does not endanger the fetus.						General knowledge
12	I feel I do not have enough knowledge to guide pregnant women regarding alcohol consumption during pregnancy.						Self-perceived competence
13	The only cause of fetal alcohol syndrome is the mother’s alcohol consumption during pregnancy.						General knowledge
14	I consistently ask pregnant women about their general alcohol drinking habits.						Professional practices
15	Drinking alcohol endangers the fetus only in the first and second trimesters of pregnancy.						General knowledge
16	One in 13 women who drink alcohol during pregnancy will give birth to a child with developmental difficulties.						General knowledge
17	“Binge” drinking (four or more drinks in about 2 h) is more dangerous to the fetus than is daily consumption of one alcoholic beverage.						Specific knowledge regarding binge drinking
18	I consistently ask pregnant women about their alcohol-drinking habits in the weeks before they learned they were pregnant.						Professional practices
